# Molecular investigation of *Blastocystis* sp. infections in wild rodents from the Inner Mongolian Autonomous Region and Liaoning province, China: High prevalence and dominance of ST4[Fn FN1]

**DOI:** 10.1051/parasite/2024031

**Published:** 2024-06-21

**Authors:** Li Liu, Long Wang, Feng Tan, Wei Zhao, Fansheng Zeng

**Affiliations:** 1 Department of Public Health and Laboratory Medicine, Yiyang Medical College Yiyang 413002 China; 2 School of Basic Medical Sciences, Wenzhou Medical University Wenzhou 325035 China

**Keywords:** *Blastocystis*, Genotype, Wild rodent, Zoonotic, Public health, China

## Abstract

Wild rodents are key carriers of various human pathogens, including *Blastocystis* spp. Our study aimed to assess the prevalence and genetic characteristics of *Blastocystis* among wild rodents in the Inner Mongolian Autonomous Region and Liaoning Province of China. From November 2023 to February 2024, 486 rodents were captured in these regions. Fresh feces were collected from the intestines of each rodent for the isolation of DNA and PCR amplification of the vertebrate cytochrome b (*cytb*) gene to identify rodent species. Subsequently, PCR analysis and sequencing of the partial small subunit of the ribosomal RNA (*rRNA*) gene were utilized to detect *Blastocystis* in all fecal samples. Of the total samples, 27.4% (133/486) were found to be *Blastocystis* positive. The results revealed the presence of four species of rodents infected with *Blastocystis*, 32.3% (63/195) in *Rattus norvegicus*, 15.1% (16/106) in *Mus musculus*, 20.2% (18/89) in *Apodemus agrarius*, and 37.5% (36/96) in *Cricetulus barabensis*. Sequence analysis confirmed the existence of five *Blastocystis* subtypes: ST1 (*n* = 4), ST2 (*n* = 2), the ST4 (*n* = 125, the dominant subtype), ST10 (*n* = 1), and a novel ST (*n* = 1). The identified zoonotic subtypes (ST1, ST2, ST4, and ST10) highlight the possible role played by wild rodents in the transmission of *Blastocystis* to humans, thereby elevating the chances of human infection. Meanwhile, the discovery of novel sequences also provides new insights into the genetic diversity of this parasite.

## Abstract (fr)

Les rongeurs sauvages sont des vecteurs clés de divers agents pathogènes humains, dont *Blastocystis* spp. Notre étude visait à évaluer la prévalence et les caractéristiques génétiques de *Blastocystis* chez les rongeurs sauvages de la région autonome de Mongolie intérieure et de la province chinoise du Liaoning. De novembre 2023 à février 2024, 486 rongeurs ont été capturés dans ces régions. Des matières fécales fraîches ont été collectées dans les intestins de chaque rongeur pour l’isolement de l’ADN et l’amplification par PCR du gène du cytochrome b des vertébrés (*cytb*) afin d’identifier les espèces de rongeurs. Par la suite, l’analyse PCR et le séquençage de la petite sous-unité partielle du gène de l’ARN ribosomal (ARNr) ont été utilisés pour détecter les *Blastocystis* dans tous les échantillons fécaux. Sur le total des échantillons, 27.4% (133/486) présentaient un résultat positif à *Blastocystis*. Les résultats ont révélé la présence de quatre espèces de rongeurs infectées par *Blastocystis*, 32.3% (63/195) chez *Rattus norvegicus*, 15.1% (16/106) chez *Mus musculus*, 20.2% (18/89) chez *Apodemus agrarius* et 37.5% (36/96) chez *Cricetulus barabensis*. L’analyse de séquence a confirmé l’existence de cinq sous-types de *Blastocystis* : ST1 (*n* = 4), ST2 (*n* = 2), ST4 (*n* = 125, le sous-type dominant), ST10 (*n* = 1) et un nouveau ST (*n* = 1). Les sous-types zoonotiques identifiés (ST1, ST2, ST4 et ST10) mettent en évidence le rôle possible joué par les rongeurs sauvages dans la transmission de *Blastocystis* à l’Homme, augmentant ainsi les risques d’infection humaine. Parallèlement, la découverte de nouvelles séquences fournit également de nouvelles informations sur la diversité génétique de ce parasite.

## Introduction

*Blastocystis* sp. is a prevalent eukaryote parasite found in the gastrointestinal tracts of both humans and animals, yet its precise pathogenicity remains enigmatic [2]. Additionally, *Blastocystis* sp. exhibits remarkable genetic diversity, encompassing 42 subtypes designated as ST1 to ST17, ST21 and ST23 to ST46, which have been documented until now [2, 9]. Notably, only 16 of these subtypes (ST1 to ST10, ST12 to ST14, ST16, ST35, and ST41) have been observed in humans, with ST1 to ST4 accounting for over 95% of the reported cases [2, 14]. Remarkably, *Blastocystis* subtypes that are typically encountered in humans were also frequently discovered in animals, such as ST4 in rodents, ST5 in pigs, ST6 and ST7 in birds, ST8 in non-human primates, and ST10 and ST14 in ruminants [3, 7]. This similarity in *Blastocystis* subtypes between humans and animals is intriguing and suggests potential cross-species transmission. Molecular characterization of *Blastocystis* subtypes present in diverse hosts, particularly those in close proximity to humans, is imperative for understanding zoonotic transmission, implications for public health, and pathogenicity.

Rodents are established reservoir hosts for a diverse array of human pathogens, *Blastocystis* being one of them [3]. Until now, *Blastocystis* infection in rodents has been reported in at least 15 countries around the world, and more than 13 subtypes have been identified in these animals, including ST1 to ST8, ST10, ST13 to ST15, and ST17 as well as some unnamed subtypes [7, 21]. Except for ST17, all the known subtypes have been reported to infect humans, further supporting the hypothesis that rodents can transmit *Blastocystis* to humans [14, 21].

In China, *Blastocystis* sp. infection has been documented in over 19 provinces and municipalities, affecting both humans and animals [13]. Until now, at least 25 subtypes (ranging from ST1 to ST17, ST21, ST23 to ST26, ST30, ST31, and ST39) have been identified [5, 13, 24–27, 30]. Notably, subtypes ST1 to ST9, ST12, and ST14 were shared by both humans and animals [5, 13, 28, 30]. Despite this knowledge, there is still a lack of data on *Blastocystis* infection in wild rodents in China [20, 23]. Liaoning Province and the Inner Mongolian Autonomous Region are primarily agricultural and pastoral regions with a significant number of domesticated animals and wild rodents. These rodents, which frequently roam on farms and animal husbandry sites, may play a crucial role in the transmission of *Blastocystis* [4]. The present study aimed to conduct a molecular survey of wild rodents from these two provinces, to determine the infection rate and genotype composition of *Blastocystis* in these animals. This information will help assess the risk of zoonotic transmission and cross-species transmission from wild rodents to other animals, providing critical baseline data for the development of effective prevention policies against *Blastocystis* infection.

## Materials and methods

### Sample collection

Between November 2023 and February 2024, a total of 486 wild rodents were collected, with 229 originating from Harqin Banner of Inner Mongolia and 257 originating from Jianping County of Liaoning Province. These rodents were captured using cage traps baited with peanut and sunflower seeds. At each designated location, around 50 cage traps were meticulously organized in a straight line, spaced at a regular distance of 5 m between each trap, to create transects. These transects were deployed precisely at 16:00 and were collected the following day at 8:00. All rodents captured were euthanized using CO_2_ inhalation and then promptly transported to the laboratory in ice boxes within 48 h. A sample of fresh feces (200 mg) was obtained from the rectum of each rodent.

### DNA extraction

A QIAamp DNA Mini Stool Kit (QIAGEN, Hilden, Germany) was utilized to extract genomic DNA from each sample, following the manufacturer’s recommended protocol. To ensure a significant quantity of DNA, the lysate’s temperature was elevated to 95 °C. Subsequently, the DNA was reconstituted in 200 μL of AE elution buffer (supplied with the kit) and stored at −20 °C prior to PCR analysis.

### PCR amplicons

The species of wild rodent was identified by amplifying the cytochrome b (*cytb*) gene of vertebrates from fecal DNA through PCR [22]. To detect *Blastocystis*, the partial *SSU rDNA* gene, which comprised 500 base pairs, was amplified using PCR. The primers, cycle conditions, and amplification system adhered strictly to the procedure outlined by Santin et al. [16]. All PCR amplifications were performed using TaKaRa Taq DNA polymerase (TaKaRa Biology, Shiga, Japan). Negative controls devoid of DNA were incorporated into each PCR assay. Agarose gel (1.5%) electrophoresis was conducted to analyze the PCR results, which were subsequently visualized using the Gel Doc EZ UV-gel imaging system (Bio-Rad Inc, Hercules, CA, USA). The colloids were stained with GelRed (Biotium Inc, Fremont, CA, USA) for enhanced visibility.

### Nucleotide sequencing and analysis

The PCR product of the anticipated fragment size (~500 bp) underwent purification using a DNA Gel Purification Kit (Sangon, Shanghai, China). Subsequently, the purified product was forwarded to Sangon Biotech (Shanghai) Co., Ltd. for bidirectional sequencing. This sequencing was executed using a BigDyeTerminator v3.1 Cycle Sequencing Kit (Applied Biosystems, Carlsbad, CA, USA), on an ABI Prism 3730 XL DNA Analyzer. After obtaining the sequences, they were carefully edited and aligned using DNASTAR Lasergene v7.1.0 and Clustal X v2.1 software. Subsequently, their genetic subtypes were determined through sequence search and alignment with reference sequences retrieved from the National Center for Biotechnology Information (NCBI) (https://www.ncbi.nlm.nih.gov/) using the basic local alignment search tool (BLAST).

### Phylogenetic analysis

To evaluate the genetic linkage between the sequences of *Blastocystis* subtypes and those archived in GenBank, a partial phylogenetic analysis was performed using the Maximum Likelihood method, employing the Tamura-3 parameter model, which was determined to be the most suitable DNA/protein model for our phylogenetic tree. To ensure the reliability of the tree, a bootstrap analysis was conducted with 1,000 replicates. The analysis was conducted within the Mega 7 software package (http://www.megasoftware.net/).

### Statistical analyses

All available data were analyzed using SPSS software version 22.0 (SPSS Inc., Chicago, IL, USA). To determine statistical significance in *Blastocystis* prevalence across rodent species and regions, the chi-square test was applied to each variable. A significance level of *p* < 0.05 was employed for all comparisons.

### Nucleotide sequence accession numbers

The nucleotide sequences of *Blastocystis* sp. found during this study has been submitted to the GenBank database with accession numbers PP504210 to PP504224.

## Results

### Rodent species identification

In this study, PCR and sequencing analysis of the *cytb* gene revealed the presence of four rodent species: *Apodemus agrarius* (*n* = 89), *Cricetulus barabensis* (*n* = 96), *Mus musculus* (*n* = 106), and *Rattus norvegicus* (*n* = 195). No additional data were gathered on these wild rodents (Table 1).

**Table 1:** Prevalence and STs of *Blastocystis* in the investigated rodents from the Inner Mongolian Autonomous Region and Liaoning province of China.

Rodent species	Inner Mongolia (Harqin Banner)		Liaoning (Jianping)		Total	
	Positive/examined (%)	*Blastocystis* ST (*n*)	Positive/examined (%)	*Blastocystis* ST (*n*)	Positive/examined (%)	*Blastocystis* ST (*n*)
*Rattus norvegicus*	46/103 (44.7)	ST4 (40), ST1 (4), ST2 (2)	17/92 (18.5)	ST4 (17)	63/195 (32.3)	ST4 (57), ST1 (4), ST2 (2)
*Mus musculus*	4/28 (14.3)	ST4 (4)	12/78 (15.4)	ST4 (12)	16/106 (15.1)	ST4 (16)
*Apodemus agrarius*	8/62 (12.9)	ST4 (7), ST10 (1)	10/27 (37.0)	ST4 (10)	18/89 (20.2)	ST4 (17), ST10 (1)
*Cricetulus barabensis*	11/36 (30.6)	ST4 (11)	25/60 (41.7)	ST4 (24), Novel ST (1)	36/96 (37.5)	ST4 (32), ST3 (4)
Total	69/229 (30.1)	ST4 (63), ST1 (4), ST2 (2), ST10 (1)	64/257 (24.9)	ST4 (63), Novel ST (1)	133/486 (27.4)	ST4 (125), ST1 (4), ST2 (2), ST10 (1), Novel ST (1)

### Infection rates of *Blastocystis* sp.

Out of 486 fecal samples, *Blastocystis* sp. was identified in 133 samples, representing a prevalence of 27.4%. Among the four rodent species studied, the infection rates were 32.3 % (63/195) in *R. norvegicus*, 15.1% (16/106) in *M. musculus*, 20.2% (18/89) in *A. agrarius*, and 37.5% (36/96) in *C. barabensis* (Table 1). Notably, statistically significant variations were observed in the incidence rates of *Blastocystis* sp. among the four rodent species (*χ*^2^ = 17.67, df = 3, *p* = 0.001). Additionally, the average infection rate of *Blastocystis* sp. among wild rodents collected from Inner Mongolia was 30.1% (69/229), which was higher than the rate of 24.9% (64/257) observed in rodents from Liaoning. However, the difference between the two infection rates was not statistically significant (*χ*^2^ = 1.67, df = 1, *p* = 0.20). Furthermore, our study indicated that the occurrence of *Blastocystis* sp. in *R. norvegicus* captured in Inner Mongolia was notably higher compared to that in Liaoning (44.7% vs 18.5%; *χ*^2^ = 15.23, df = 1, *p* < 0.01). Conversely, the prevalence of *Blastocystis* sp. among *A. agrarius* was notably higher in Liaoning than in Inner Mongolia (37.0% vs 12.9%; *χ*^2^ = 6.79, df = 1, *p* = 0.01). Although the infection rates of the other two species of wild rodents were higher in Liaoning than in Inner Mongolia, these differences were not statistically significant (41.7% vs 30.6% for *C. barabensis* and 15.4% vs 14.3%v for *M. musculus*; *p* > 0.05) (Table 1).

### Sequencing of PCR amplicons

The subtypes of *Blastocystis* were detected by sequencing each of the 133 PCR positive products. Five subtypes, including four known named as ST1, ST2, ST4 and ST10, and one novel ST were identified, without mixed subtype infections. ST4 subtype was the most prevalent, resulting in 94.0% (125/133) of the *Blastocystis* positive samples. This subtype was found in all four rodent species included in the survey. The remaining subtypes had a low frequency with ST1 being identified in four *R. norvegicus*; ST2 also in two *R. norvegicus*; ST10 in an *A. agrarius*; and the novel ST in a *C. barabensis* (Table 1). Furthermore, the composition of subtypes varied between different regions, specifically ST1, ST2 and ST10 in Inner Mongolia (Harqin Banner), and the novel ST in Liaoning (Jianping).

### Genetic diversity of *Blastocystis* subtypes

**Figure 1:**
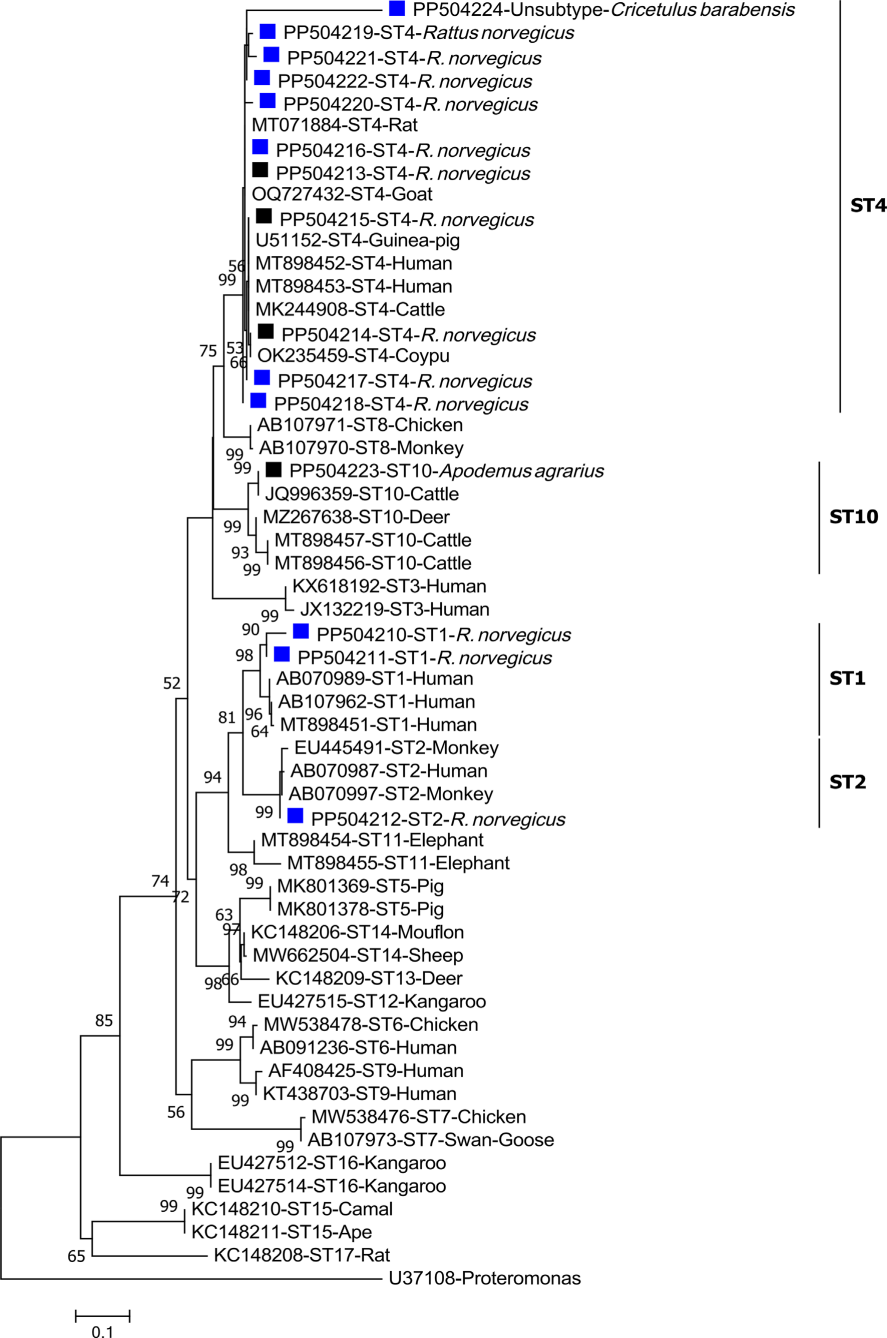
Phylogenetic relationships among the *Blastocystis* sequences identified in this study were analyzed alongside previously stored sequences in GenBank, using the maximum likelihood method. This approach used the Tamura-3 parameter model within the Mega 7 software package. The percentages on the branches represent the bootstrap values obtained from 1,000 replicates. These sequences were uniquely identified by their accession number, host origin, and ST designation. The novel and known sequences identified in this study are marked with blue and black squares, respectively.

Of the 133 identified sequences, 15 representative sequences were observed, with 10 sequences designated as ST4 (labeled as ST4-1 to ST4-10 for descriptive purposes), two sequences designated as ST1 (ST1-1 and ST1-2), one sequence designated as ST10, one sequence designated as ST2, and one sequence belonging to a previously unknown ST. Among the 10 sequences of ST4, three representative sequences have been previously described: ST4-1 (PP504213; *n* = 90), which is 100% identical to the ST4 sequence MT071884 from Chinese laboratory rat and 30 other sequences in GenBank; ST4-2 (PP504214; *n* = 18), which is 100% identical to the ST4 sequence from Chinese coypu (OK235459) and 6 other sequences in GenBank; and ST4-3 ((PP504215; *n* = 10), which is identical to the ST4 sequences identified in cattle from the United States (MK244908) and humans from Spain (MT898453). The remaining seven representative sequences of ST4 (ST4-4 to ST4-10) each appeared in a single sample, have not been previously described, and have a similarity 98.6–99.8% with the closest known sequences (Table 2). Both the two representative sequences of ST1 are novel: ST1-1 (PP504210; *n* = 3) and ST1-2 (PP504211; *n* = 1) have 96.8% and 99.3% similarity to KR26289 and KR262915, respectively, both were identified in long-tailed macaque from China. Two samples share an ST2 sequence (PP504212) that differs from the sequence KU719534 of ST2 identified in humans from Iran by a single base (G to A at 422 site). The ST10 sequence (PP504223) is 100% identical to the sequence JQ996359 of ST10 from cattle in the United States. The novel ST sequence (PP504224) has a maximum similarity of only 86.9% with a known *Blastocystis* ST4 sequence (OQ727432), which was identified in a goat from China. The nucleotide sequences of *Blastocystis* sp. subtypes discovered in this study were grouped into their respective evolutionary branches, with the novel ST sequence forming a separate branch in the phylogenetic tree (Fig. 1).

**Table 2:** Similarity analysis of the *Blastocystis* sequences obtained in the present study.

Subtypes (*n*)	Accession no.	Ref accession numbers#/Identities/Nucleotide positions
ST1-1 (3)	PP504210	KR26289/96.8%/Long-tailed macaque/China
ST1-2 (1)	PP504211	KR262915/99.3%/Long-tailed macaque/China
ST2 (2)	PP504212	KU719534/99.8%/Human/Iran
ST4-1 (90)	PP504213	MT071884/100%/Laboratory rat/China
ST4-2 (18)	PP504214	OK235459/100%/Coypu/China
ST4-3 (10)	PP504215	MK244908/100%/Cattle/the UAS
ST4-4 (1)	PP504216	ON834468/99.5%/Bear/China
ST4-5 (1)	PP504217	OQ727432/99.8%/Goat/China
ST4-6 (1)	PP504218	OQ727432/99.8%/Goat/China
ST4-7 (1)	PP504219	OQ727432/98.6%/Goat/China
ST4-8 (1)	PP504220	OQ727432/99.1%/Goat/China
ST4-9 (1)	PP504221	OQ727432/99.1%/Goat/China
ST4-10 (1)	PP504222	OQ727432/99.8%/Goat/China
ST10 (1)	PP504223	JQ996359/100%/Cattle/the UAS
New subtype (1)	PP504224	OQ727432/86.9%/Goat/China

# Sequence displaying the highest degree of similarity, or with one of the highest similarities.

## Discussion

This study presents the first investigation of *Blastocystis* among wild rodents in the Inner Mongolia Autonomous Region and Liaoning Province, revealing an average infection rate of 27.4%. Globally, *Blastocystis* infection has been reported in rodents across 15 countries, with infection rates ranging from 3.0% to 100% [7]. Notably, the infection rate among wild rodents (30.5%) is significantly higher than that among domesticated (12.3%), laboratory (8.2%), and pet rodents (7.7%) [10]. In China, the majority of studies have been conducted on domesticated, pet, or laboratory rodents, which generally enjoy better hygiene conditions, explaining their lower infection rates [29]. Furthermore, it is crucial to highlight that out of the 15 countries, 80% have conducted only one study, and these studies involve a relatively small number of rodents [7]. Therefore, caution must be exercised when interpreting the true prevalence of rodent *Blastocystis* infection in these countries. In order to obtain a comprehensive understanding of the *Blastocystis* infection prevalence among rodents, it is imperative to expand the scope and depth of existing studies. This includes increasing the number of countries represented, expanding the range of rodent species studied, and increasing the sample sizes to ensure more statistically robust results. Additionally, it is crucial to use standardized methods and protocols to ensure consistency and comparability across studies.

This study found four known subtypes of *Blastocystis* sp. (ST1, ST2, ST4, and ST10), with ST4 being the most prevalent subtype found in 94% of the *Blastocystis*-positive animal samples. Previous studies have demonstrated that ST4 is found in over 20 rodent species globally; therefore, this subtype may have evolved to infect rodents [29]. ST4 is also prevalent in humans worldwide, with a global occurrence rate of 5.9% and Europe accounts for a significant proportion of human cases, reaching up to 19.8% [14]. In China, cases of ST4 infection in humans have been reported in multiple provinces including Zhejiang, Henan, and Yunnan [5, 28]. Meanwhile, ST4 has also been identified in various rodent species in China, including coypus, bamboo rats, porcupines, civets, and brown rats [29]. Additionally, it has been detected in domesticated animals such as cows and goats, captive wildlife like Alpine musk deer and black bears, as well as birds like domestic pigeons and swans from China [29, 31, 32]. Although there are currently no reports of *Blastocystis* detection in China’s water environment, previous studies have identified ST4 in various water sources in other Asian countries, suggesting that ST4 can contaminate water sources through rodents, and may infect humans and other animals [12, 15]. Therefore, future research should adopt a One Health approach to explore the infection/carriage status of *Blastocystis* in humans, animals, and the environment, aiming to better understand its transmission routes and sources of infection.

In this study, ST1 and ST2 were identified in four and two wild rodents surveyed, respectively, further highlighting the role of wild rodents in transmitting *Blastocystis* to humans. Notably, ST1 and ST2 rank second and third in causing *Blastocystis* infections in humans, with an estimated 27.2% and 14.8% of human cases attributed to them, respectively [14]. Worldwide, animals from diverse species have been found to carry ST1 and ST2 [7]. In China, ST1 has been identified in foxes, civets, birds, bears, non-human primates, pigs, and dogs [5]. Likewise, ST2 has been observed primarily in non-human primates, bears, and certain captive wild animals [5]. Although only 1.3% of the rodents in this study were identified as infected with ST1 and ST2, their potential as sources of *Blastocystis* infection for humans and other animals cannot be overlooked.

In the current study, ST10 was identified in an *A. agrarius*. Globally, it is the most frequently reported subtype in goats, sheep, and cattle, and is also widely prevalent in companion animals such as cats, dogs, and horses [1, 17–19]. Additionally, ST10 has been detected in other animals including pigs, deer, bears, antelopes, chickens, swans, and wild birds [6, 7, 31]. Although human infections with ST10 have been relatively rare, reports of this subtype have emerged in Senegalese school children and Egyptians since 2020 [8, 11]. Recently, a study conducted in Vietnam revealed that ST10 ranks second highest in humans after ST3 [12]. This is the first identification of ST10 in *A. agrarius*, broadening the range of potential hosts for this subtype and highlighting the potential transmission between humans and other animals, particularly cattle, goats, and sheep. Notably, this subtype has been reported to dominate in cattle, goats, and sheep in the same geographical region as the study [24, 27]. Nevertheless, given the limited number of infected animals (only one), we cannot say definitively that the rodent is a true host for this subtype rather than a mechanical carrier. Therefore, it is particularly important to conduct more research on rodents carrying *Blastocystis*, so that we can more comprehensively understand their true situation, including infection rates and subtype distribution.

This study has uncovered a novel sequence, exhibiting a maximum similarity of 86.9% to the currently known *Blastocystis* subtype sequences. Nevertheless, owing to the absence of the full-length *SSU rRNA* gene sequence for this potential novel subtype, we are unable to designate it as a new subtype (potentially ST47) based on the latest subtype naming guidelines [20]. Therefore, we have designated it as a novel ST. Obtaining a full-length reference sequence from samples harboring this potential novel ST in the future will aid us in clarifying its categorical status and epidemiological characteristics. Additionally, we anticipate that further research, particularly among rodent populations given their vast numbers, will lead to the discovery of more subtypes.

## Conclusions

This study demonstrated a high prevalence of *Blastocystis* among four wild rodent species in the Inner Mongolia Autonomous Region and Liaoning Province of China. Notably, four known potentially zoonotic subtypes were identified, with ST4 being dominant. These observations imply that wild rodents could potentially act as reservoirs for human *Blastocystis* infection. Furthermore, the first report of ST10 in rodents, along with the discovery of a putative novel ST and several unique sequences, provides valuable insights into the genetic diversity of *Blastocystis*.
